# A review of hydrogels used in endoscopic submucosal dissection for intraoperative submucosal cushions and postoperative management

**DOI:** 10.1093/rb/rbad064

**Published:** 2023-06-22

**Authors:** Zhihong Chen, Jie Ding, Chengheng Wu, Dan Wei, Jing Sun, Hongsong Fan, Zhenzhen Guo

**Affiliations:** National Engineering Research Center for Biomaterials, College of Biomedical Engineering, Sichuan University, Chengdu, Sichuan 610064, China; National Engineering Research Center for Biomaterials, College of Biomedical Engineering, Sichuan University, Chengdu, Sichuan 610064, China; National Engineering Research Center for Biomaterials, College of Biomedical Engineering, Sichuan University, Chengdu, Sichuan 610064, China; Institute of Regulatory Science for Medical Devices, Sichuan University, Chengdu, Sichuan 610064, China; National Engineering Research Center for Biomaterials, College of Biomedical Engineering, Sichuan University, Chengdu, Sichuan 610064, China; National Engineering Research Center for Biomaterials, College of Biomedical Engineering, Sichuan University, Chengdu, Sichuan 610064, China; National Engineering Research Center for Biomaterials, College of Biomedical Engineering, Sichuan University, Chengdu, Sichuan 610064, China; Department of Gastroenterology, Sichuan Province People's Hospital, University of Electronic Science and Technology of China, Chengdu, Sichuan 610072, China

**Keywords:** hydrogels, biopolymer, gastrointestinal cancers, submucosal injected materials, postoperative management

## Abstract

Endoscopic submucosal dissection (ESD) has been clinically proved to have prominent advantages in the treatment of early gastrointestinal cancers over traditional surgery, including less trauma, fewer complications, a quicker recovery and lower costs. During the procedure of ESD, appropriate and multifunctional submucosal injected materials (SIMs) as submucosal cushions play an important role, however, even with many advances in design strategies of SIMs over the past decades, the performance of the submucosal cushions with postoperative management function seems to be still unsatisfactory. In this review, we gave a brief historical recount about the clinical development of SIMs, then some common applications of hydrogels used as SIMs in ESD were summarized, while an account of the universal challenges during ESD procedure was also outlined. Going one step further, some cutting-edge functional strategies of hydrogels for novel applications in ESD were exhibited. Finally, we concluded the advantages of hydrogels as SIMs for ESD as well as the treatment dilemma clinicians faced when it comes to deeply infiltrated lesions, some technical perspectives about linking the clinical demand with commercial supply were also proposed. Encompassing the basic elements of SIMs used in ESD surgery and the corresponding postoperative management requirements, this review could be a good reference for relevant practitioners in expanding the research horizon and improving the well-being index of patients.

## Graphical Abstract

**Figure:**
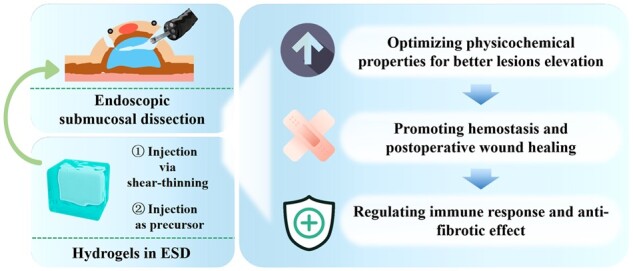

## Introduction

Cancer is one of the major causes of death and a significant impediment to extending life expectancy worldwide. According to GLOBOCAN database for the year 2020, gastrointestinal (GI) cancers, including esophageal, stomach, colorectal cancers, contributed to 18.5% of global cancer incidence and 22.4% of all cancer-related fatalities, with an estimated 3.57 million new cases and 2.22 million deaths [1]. The clinical stage of GI cancers has a significant impact on their prognosis and rate of regression. For individuals with early-stage GI cancers that do not carry a risk of lymph node metastases, endoscopic resection is the preferred course of action. Endoscopic treatment of early GI tumors has prominent advantages over traditional surgery, including less trauma, fewer complications, a quicker recovery and lower costs [2]. This allows surgical-level efficacy to be achieved and patients’ quality of life to be improved while still preserving the structural integrity of the GI tract [3–5].

Endoscopic submucosal dissection (ESD) is a cutting-edge method for the early treatment of GI cancers due to its ease of use and safety [3]. In the process, submucosal injection can significantly aid in the implementation of quick and secure endoscopic procedures by creating a fluid cushion. An ideal submucosal fluid cushion can elevate the mucosa to be resected and isolate it from the muscular layer, accordingly restricting the high-frequently current in the mucosal layer and increasing technical feasibility. Thus, thermal injury and the danger of perforation and bleeding are decreased while *en bloc* resection is enabled.

Varieties of submucosal injection materials (SIMs) have been applied in ESD, including normal and hypertonic saline, dextrose solution, glycerol, hydroxypropyl methylcellulose, sodium hyaluronate and others [6–10]. However, all of these products have a number of flaws and are incapable of promoting wound healing, which falls far short of therapeutic requirements. In some researches, additional intraoperative sprays of bioprotein gels and mucosal protectants are employed to help postoperative wound healing in severe lesions, which complicates the operation and lacks evidence of its effectiveness [11–13].

Herein, we outline some of the most popular submucosal injection agents, contrast them while taking into both their benefits and drawbacks, and look into the distance their development direction.

## The standard procedures and the proposed SIMs of ESD

**Figure 1.**
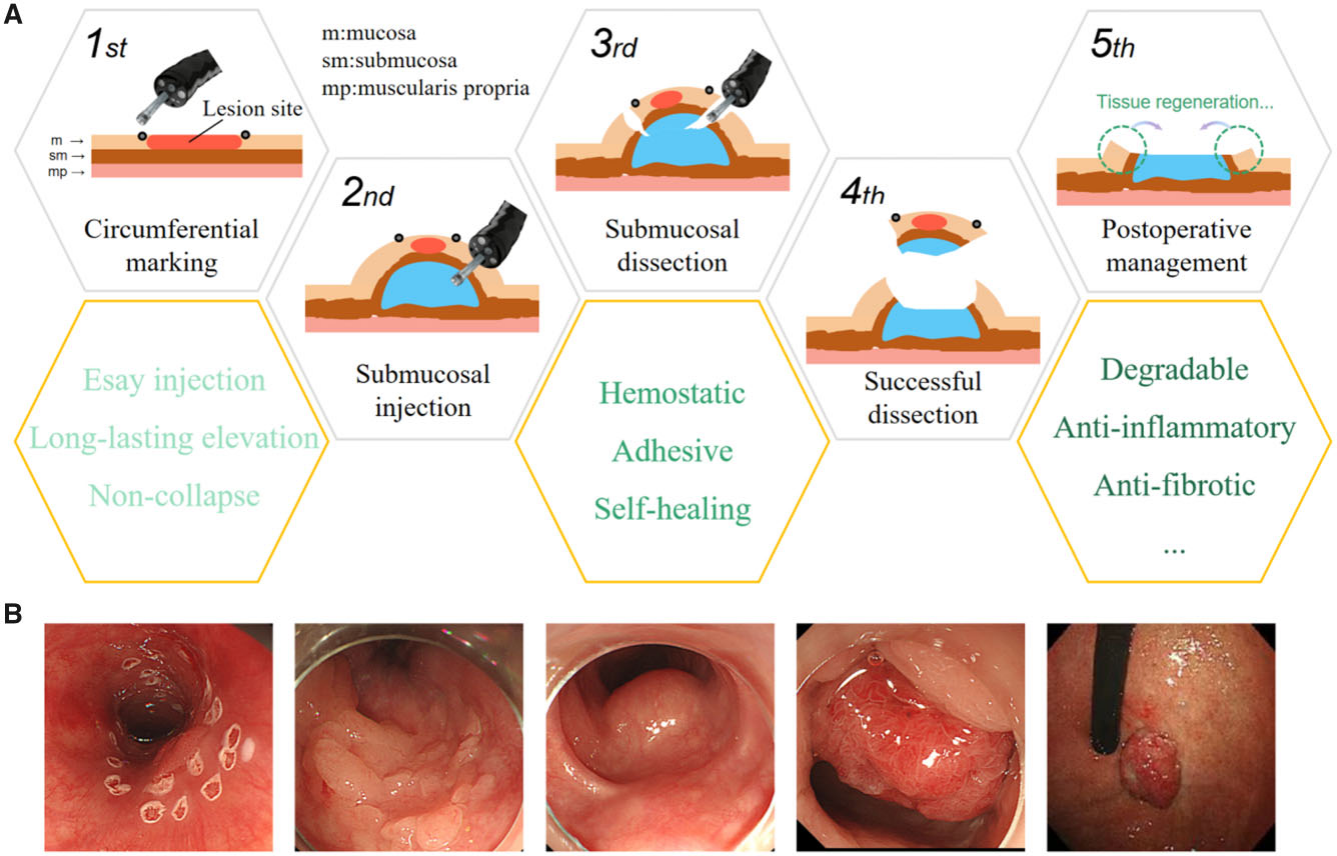
(**A**) A schematic of the whole process of ESD surgery and the corresponding performance requirements for SIMs. (**B**) Some digital images of the GI lesions needed to be treated with ESD.

The standard steps of ESD procedures typically include: circumferential marking after defining the lesion border, submucosal injection at various locations surrounding the lesion, circumferential dissection, submucosal dissection and wound management (Fig. 1A) [4]. Among them, intraoperative submucosal injection to elevate lesions is the key to the safe and successful ESD, since it could not only separate mucosal layer and muscular layer, but also indicate the depth of invasion. If the lesion is heavily infiltrated or poorly raised following submucosal injection, the risk of intraoperative and postoperative bleeding, perforation and other severe consequences is also markedly enhanced.

For lesions where a quick ESD is anticipated, such as neuroendocrine neoplasm in rectum or small lesions in the esophagus, separate submucosal injections are frequently required in various areas of the lesion in order to lift the entire lesion intact. Figure 1B shows some typical photographs of clinical lesions requiring surgical dissection. Due to the diffuse nature of existing common fluid cushion, submucosal injections can also be carried out in stages for lesions when the operation is anticipated to take longer. After performing the submucosal injection of the cushion in a portion of the lesion and dissecting that portion of the lesion, the cushion is next submucosal injected in the remaining portion of the lesion. Theoretically, to effectively elevate lesions, the submucosal agents should be fluid at the time of injection, then maintain a fixed shape without any change in mechanical properties for a certain period of time after injection into the target area. Thus, the ideal material for submucosal injection in ESD for lesion elevation should satisfy the following characteristics: safety, good syringeability, *in situ* curing retention ability, certain mechanical strength for height retention.

Actually, early ESD SIMs mainly concentrated on their physical requirement for surgical manipulation. However, there is also a chance of delayed bleeding, postoperative perforation and stenosis, in addition to intraoperative complications [3]. And postoperative bleeding may appear up to 2 weeks after ESD surgery especially when minor and easily overlooked muscular layer injuries occur [14]. After endoscopic treatment, persistent inflammation is exacerbated by infection and varied physicochemical stimulations, resulting in delayed healing of the wounds and bleeding [15]. Moreover, muscularis fissure occurred in ESD procedure could develop to perforation due to the corrosive effect of digestive fluid [16]. Furthermore, during the healing process of ESD wounds, an overactive inflammatory response may cause submucosal fibrosis to proliferate uncontrollably and scar tissue to constrict, ending with stenosis of the digestive tract, especially the esophagus [17]. Consequently, biological functions of materials, such as the promotion of postoperative wound healing, the prevention of postoperative stenosis and antibacterial property, are also required for clinical purpose.

Hence, if the submucosal injection matrix contains antibacterial, wound-healable and anti-fibrosis properties, the post-operation complications can be significantly reduced. In other word, the rational materials for submucosal injection in ESD should possess both the physical abilities mentioned above to elevate the lesion and the biological capacities for repair.

## Three stages of SIMs’ development

**Figure 2.**
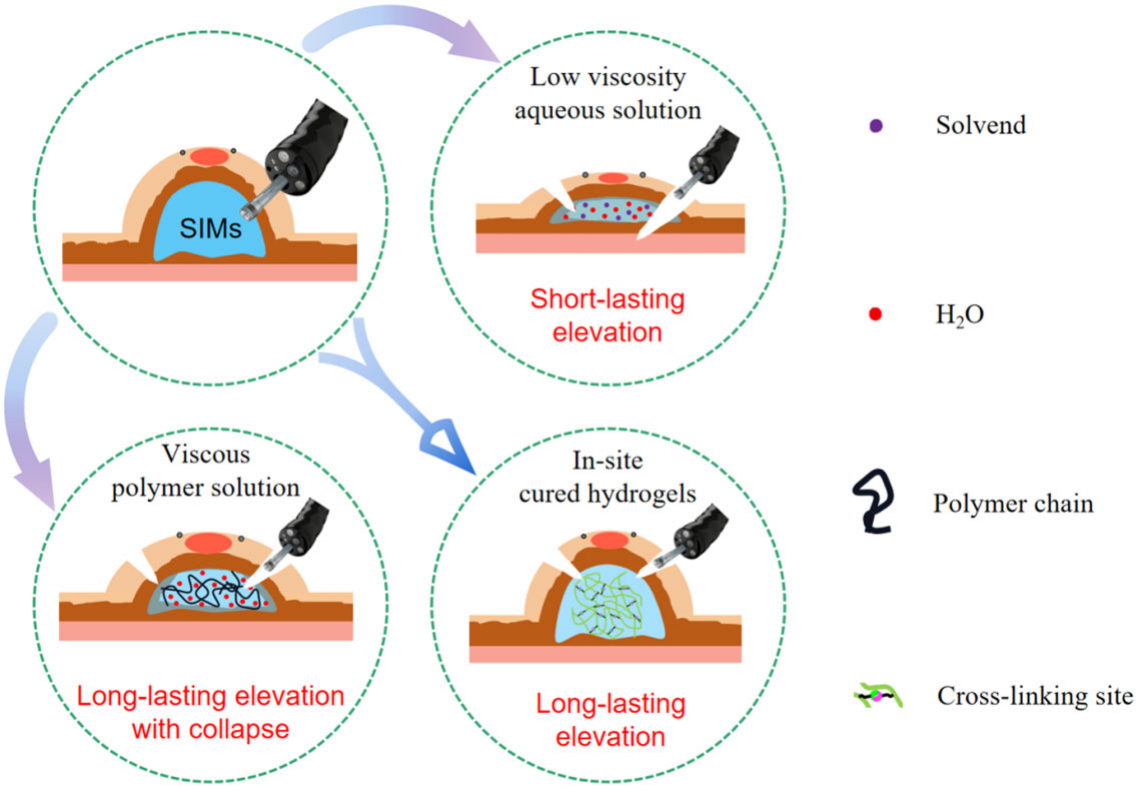
A comparison diagram of three commonly used SIMs: low-viscosity aqueous solution, viscous polymer solution and in-site cured hydrogels.

Since ESD technology was proposed, biomaterials for ESD use have gone through three stages, resulting in three types of SIMs. As far as we know, there are three major types of SIMs: low-viscosity aqueous solution, viscous polymer solution and in-site cured hydrogels. Below is the corresponding schematic diagram (Fig. 2) of above three materials.

### Low-viscosity aqueous solution for early and general clinical use

Based on basic mucosal separation requirements, and due to its strong biocompatibility, wide accessibility, inexpensive cost and inappreciable toxic effect, physiological saline became the most popular elevation matrix for ESD [18]. However, its elevating effect on the lesion is minimal since saline itself is totally flowable and not mechanically supportive. Additionally, physiological saline rapidly infiltrates into the surrounding tissues during surgery, making it difficult to maintain the height of submucosal cushion for a longer duration, which requires repeated and numerous supplemental injections.

In addition, because of their biosafety, other fluids comparable to saline are also employed for the purpose of creating higher lesion lifting such as hypertonic saline, dextrose water and glycerin [7]. But Fujishiro *et al.* [7] had demonstrated that the superiority of hypertonic solutions over regular saline for causing and maintaining long-term mucosal elevation was not clearly demonstrated. Not only that, hypertonic solutions may cause localized histopathological injury and trigger inflammatory reaction in local area through osmotic pressure gradients when their ion concentration is badly misaligned with that of intracellular ions [9]. Furthermore, due to the native low viscosity of these solvents, repeating injection during ESD surgery is needed, thus the loose connective tissue in the submucosa will be stretched and the elevation effect of supplemental injection in later period will be poor. As a result, in theory, the use of SIMs with higher viscosity will lead to better elevation effect for a complete and smooth ESD surgery.

### Viscous polymer solution for better elevation effect

**Figure 3.**
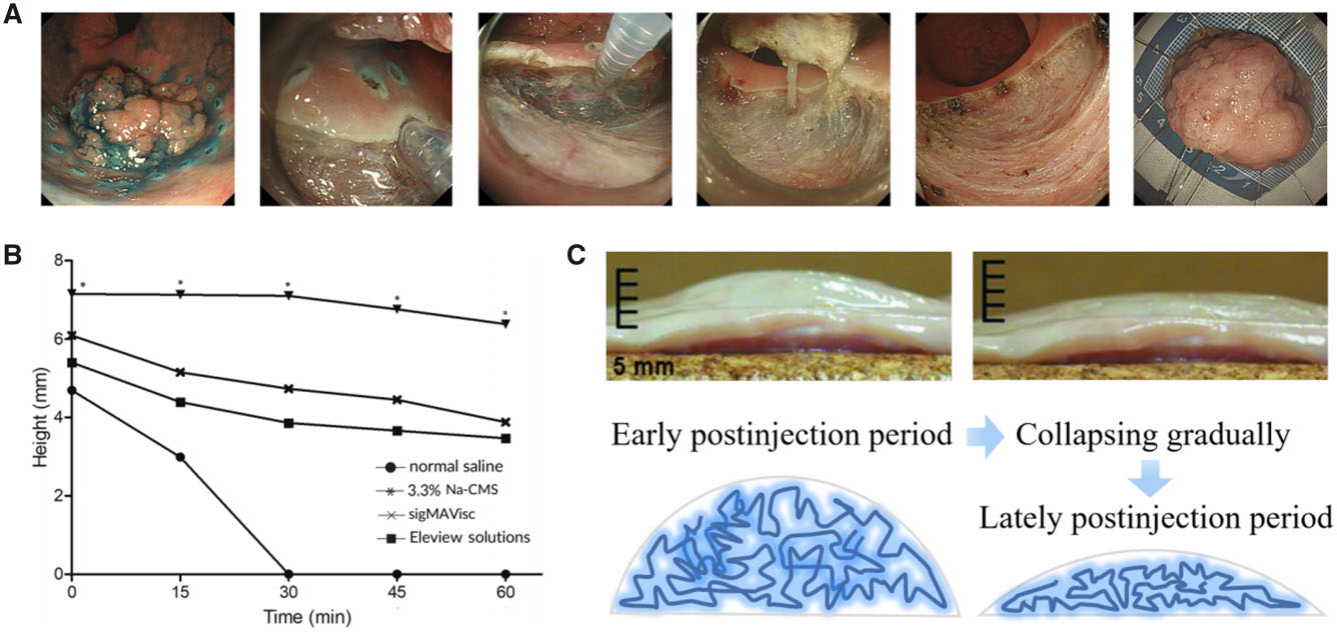
(**A**) A set of clinical diagrams of a typical ESD procedure using sodium hyaluronate solution (reproduced with permission from John Wiley and Sons). (**B**) Chronological changes in the mucosal elevation following injection of normal saline, 3.3% Na-CMS, sigMAVisc or Eleview solutions. *P <* *0.05 compared with normal saline solution, Eleview, sigMAVisc groups, respectively, at each time point (0, 15, 30, 45, 60 min) (reproduced with permission from John Wiley and Sons). (**C**) Schematic diagram of viscous polymer solution collapse over time (reproduced with permission from Elsevier).

The viscosity of aqueous or hypertonic small-molecule solutions is very low, thus resulting in a lack of long-term mucosal elevation effects. Since polymers are long chains of entangled molecules, which creates a resistance to flow and increases the viscosity of the solution, the fluidity of the polymer solution for use in ESD injection is preserved. Therefore, viscous polymer solution is expected to have better effects than non-viscous normal saline in elevating mucous layer and improving maintenance time before collapse. Consequently, fluids with specified viscosity, such as sodium hyaluronate [19, 20], sodium carboxymethyl starch [21] and fibrin glue [22], have been employed for submucosal injection. Figure 3A vividly shows a set of clinical diagrams of a typical ESD procedure using sodium hyaluronate. However, there are also many limitations and problems in the clinical use of the above solutions.

Aliaga Ramos *et al*. [19] compiled a set of clinical data on ESD, which came from 78 patients using sodium hyaluronate for submucosal injection. The *en bloc* resection rate was 96.1%, and the curative resection rate for epithelial lesions was 83.8%, while five instances (6.3%) experiencing adverse outcomes such as delayed bleeding and perforation. In some other cases, sodium hyaluronate solution with high viscosity was used to show satisfaction in elevating mucous layer, whereas its fluid state could lead repeated intraoperative injections which undoubtedly increased the cost due to that sodium hyaluronate is relatively expensive [20]. Sodium carboxymethyl starch (Na-CMS) designed by Chen *et al*. [21] has a relatively high viscosity, resulting in higher submucosal elevation, lower required injection volume, shorter resection time and less severe hemorrhage than the control group in ESD (Fig. 3B), but problems in this study such as too small sample size and unprecise setting of control group may hinder the clinical application.

Clinical experience shows that submucosal fluid leakage from the injection site is inevitable when normal saline or sodium hyaluronate is used for submucosal injection. To solve the issues, Takao *et al*. [22] have proved that fibrin glue, a blood-derived product that contains two component solutions: fibrinogen and thrombin solution, could be used as an ESD submucosal injection agent because of these two components are totally biocompatible and they could create a stable clot when mixed which would not be absorbed rapidly into the surrounding issue. However, it should be taken into account that biological products like fibrin glue come at a cost and may be contaminated, increasing the risk of infection [23, 24].

More and more clinical cases of ESD show that a series of intraoperative and postoperative problems such as perforation and delayed bleeding caused by collapse of elevated mucosal with the increase of surgical time are still difficult to be solved when polymer solution is applied (Fig. 3C) [25]. The fundamental reason is that there is an inherent tradeoff between the viscosity required to elevate lesion and the fluidity required for injection when considering the optimum elevation height and time. And this dilemma will lead the viscous polymer solution as submucosal cushion to collapse over time, thus leading a series of problems during ESD surgery.

### In-site cured hydrogels

Hydrogels, known as a water-rich, 3D polymeric network, are extensively employed as delivery systems for drugs and cells or as scaffolds for tissue engineering [26–30], due to their native controllable gelation property from liquid to solid, besides the excellent permeability, biocompatibility and biodegradation.

**Figure 4.**
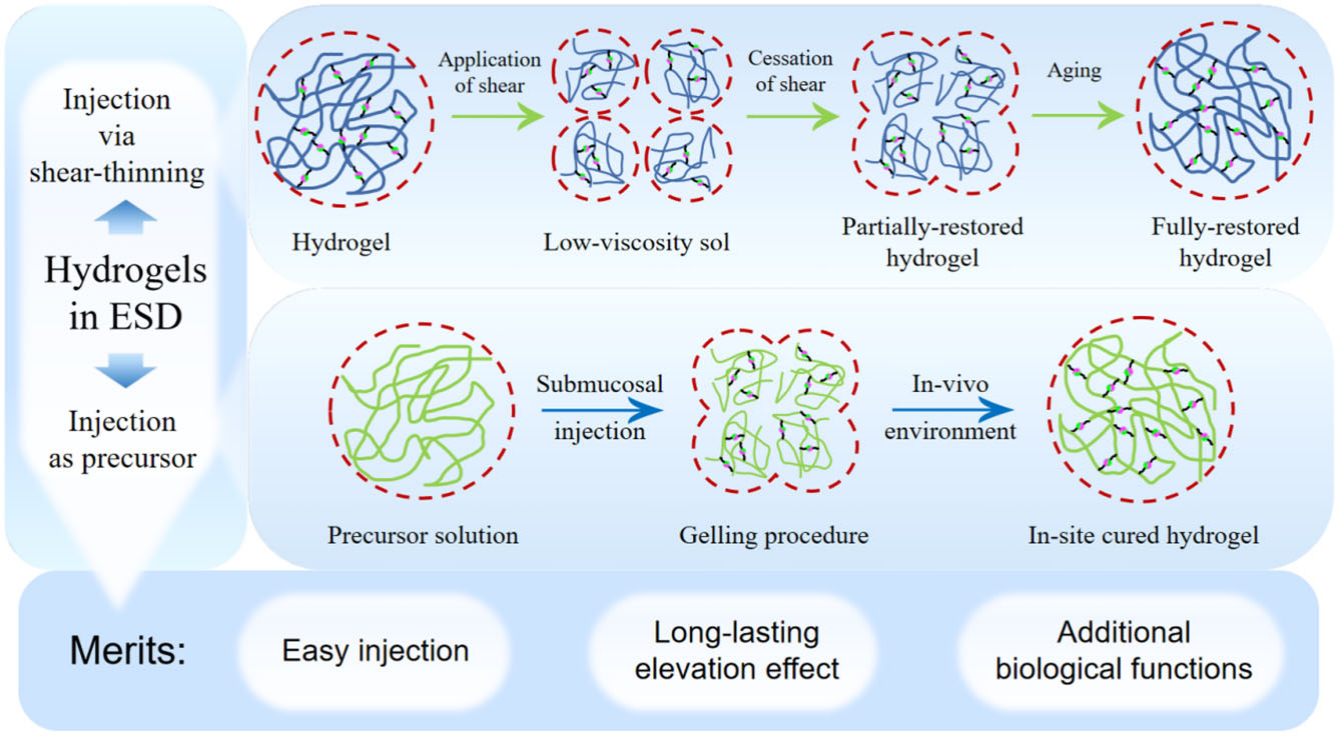
Two ways of using hydrogels in ESD and their potential advantages.

In the gelation and degradation course of hydrogels, cross-linking plays a crucial role, which is a stabilizing procedure resulting in the multi-dimensional expansion of polymeric chains, culminating in network architectures. The reason is that polymer is a kind of materials with long molecular chains. After cross-linking, many polymer chains will crisscross together and form many nanoscale grids. And most of the polymers contain sufficient hydrophilic groups, which will cause a large number of water molecules to flow into above nanoscale grids through surface adsorption, capillary action or diffusion. With the continuous increase of water molecules, the molecular chain of polymer will be crowded out and then lead to the corresponding multi-directional expansion. Then hydrogels are generated into stable structures from their original forms [31, 32]. In short, hydrogel curing is a process in which fluid liquid state converts into cross-linked solid state, which is very similar to the injection process for SIMs in ESD: flowable during injection and sustainable solid after injection. As a consequence, in ESD procedure, hydrogel precursor solution could be injected submucosally in the form of liquid, then gelate in-site to form solid-state hydrogels in physiological circumstances such as temperature, pH, enzyme and exogenic photo-induced polymerization [33–43], thus providing mucosal elevation effects without collapse as well as avoiding the repeated injection during surgery procedure. What is more, partial hydrogels might be directly injected into the submucosa using a tiny endoscopic needle, taking advantage of the shear-thinning property, and concurrently produce a durable submucosal cushion. The principles of above two hydrogels used in ESD are shown in Fig. 4.

For above reasons, in-site cured hydrogels create minimally invasive methods that offer many advantages over the use of preformed elevating agents such as conformability in any wound scenario, convenience of application, and improvement of patients’ compliance and comfort. Specially, the retained hydrogels could biodegrade safely in human tissue after a period time, ensuring long-term security [33, 44]. Therefore, development of an optimal in-site cured hydrogel for submucosal injection in ESD is the new trend of current research for ESD.

## Application of hydrogels in ESD

In clinical application, especially for the possible requirement of postoperative management, the biocompatibility and bioactivity of hydrogels play a key role besides the injection properties, even directly affect the success of ESD surgery. Hence, we herein classify them according to the source of hydrogel precursor, which can be divided into two classes, synthetic polymer hydrogels and natural biopolymer hydrogels.

### Synthetic polymer hydrogels in ESD

Synthetic polymer hydrogels are usually cross-linked by synthetic hydrophilic polymers through physical or chemical cross-linking action, including alcohols, acrylic acid and its derivatives (polyacrylic acid, polymethacrylic acid, polyacrylamide, poly-N-polyacrylamide etc.), which are usually prepared by polymerization reactions including step polymerization, free-radical polymerization, ionic polymerization (anionic polymerization, cationic polymerization), coordination polymerization, ring-opening polymerization and copolymerization. In general, the physical characteristics of synthetic hydrophilic polymers are easy to be adjusted.

Injectable hydrogels for ESD use require the gel precursor to be injected fluidly into the surgical site and in-site gelate to form a solid support. Therefore, responsive polymers with biocompatible and controllable gelation during surgery are preferred for ESD.

**Figure 5.**
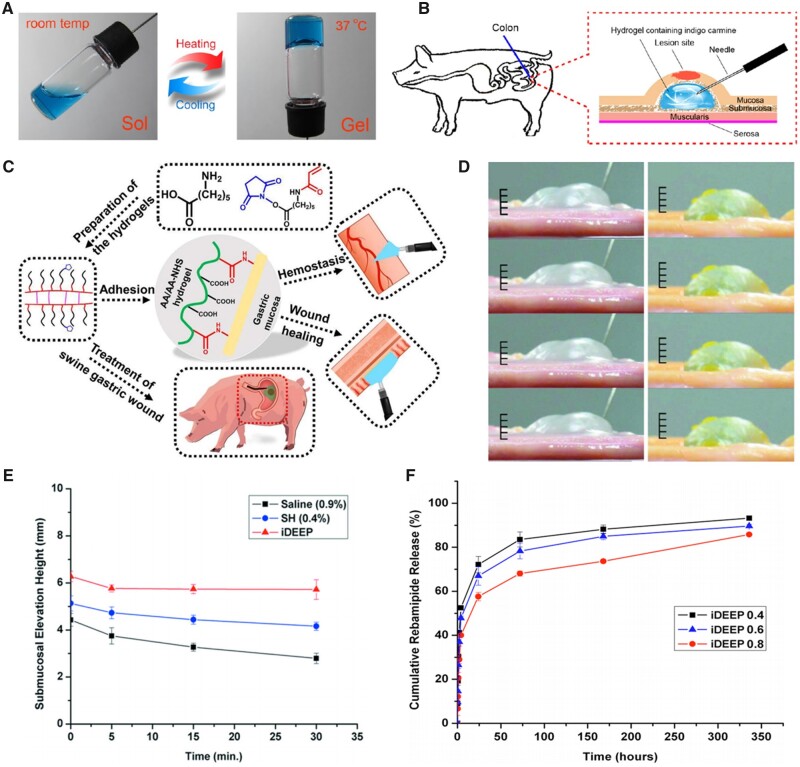
Various synthetic polymer hydrogels used in ESD. (**A**) Photographs of the PLGA-PEG-PLGA solution (15 wt% in normal saline) containing indigo carmine exhibiting a sol at room temperature and a gel after heated to body temperature. The indigo carmine was used as the color agent for visualization of the procedure (source article published under open access, CC-by license). (**B**) Schematic diagram showing the creation of submucosal fluid cushion following the submucosal injection of thermosensitive hydrogel (source article published under open access, CC-by license). (**C**) Schematic representation of the synthesis and applications of the AA/AA-NHS hydrogel (source article published under open access, CC-by license). (**D**) Photographic images depicting the chronological changes in the submucosal elevation of sodium hyaluronate (left), and injectable drug eluting elastomeric polymer (iDEEP) hydrogels (right) from top to bottom in turn 1, 5, 15 and 30 min after injection using porcine gastric samples *ex vivo* (reproduced with permission from Elsevier). (**E**) Graphical representation of the chronological changes in the submucosal elevation (reproduced with permission from Elsevier). (**F**) *In vitro* rebamipide release profiles from injectable iDEEP hydrogels (reproduced with permission from Elsevier).

Thermal cross-linking is a simple and controllable cross-linking method, therefore, as a typical synthetic polymer, hydrophilic poly(ethylene glycol) (PEG) likely to produce thermosensitive hydrogels becomes one of the key ingredients as SIMs in ESD. Actually, the thermoreversible poly(lactic acid-co-glycolic acid)–PEG–poly(lactic acid-co-glycolic acid) (PLGA–PEG–PLGA) hydrogel has been reported for application in ESD as a novel SIM to create fluid cushion, whose thermal response performance could be optimized by adjusting the block ratio (Fig. 5A). In the animal models, the PLGA–PEG–PLGA thermosensitive-hydrogel was conveniently injected into submucosa as a sol at room temperature, subsequently spontaneous in-site gelation at body temperature and elevate the mucosa successfully (Fig. 5B). The use of PLGA–PEG–PLGA hydrogel significantly shortened the procedure’s duration, made it easier, and decreased the risk of complications [45, 46]. However, due to the physical cross-linking catalyzed by hydrophobic interactions, the thermosensitive hydrogels frequently have weak gel strength, which may restrict their further applications in major and prolonged operations. Furthermore, when it is clinically applied in ESD procedure, due to the operator’s proficiency, the complexity of the operation site and other factors, once body temperature is achieved in the long injection tubing, the thermosensitive hydrogels may clog inside the long tube prematurely, making smooth injection more challenging.

Another class of synthetic polymers with pH-sensitive properties have also been used in ESD in recent years. The structural characteristics of pH-sensitive polymers could be drastically changed at pH levels above and below the prescribed range, leading mutation of the hydrodynamic properties of the polymers. He *et al*. [47] have developed a variety of injectable pH-responsive hydrogels based on acryloyl-6-aminocaproic acid (AA) and AA-g-N-hydroxysuccinimide (AA-NHS), which showed significant promise as endoscopic sprayable bioadhesive materials to effectively halt bleeding and accelerate the healing process. The AA/AA-NHS hydrogels were synthesized by mixing the AA and AA-NHS solutions together at room temperature with N,N′-methylenebisacrylamide as a cross-linker to form a network of hydrogels via free-radical polymerization. It should be noted that besides the common elevating effect, these hydrogels displayed autonomous and effective self-healing, hemostatic characteristics, an appropriate gelation duration and improved adhesive strength (Fig. 5C). In the swine model, these hydrogels showed tremendous promise for treating stomach wounds after endoscopic therapies. However, in this report, the hydrogels were all sprayed directly over the wound surface without submucosal injection, and corresponding elevating effect during ESD was not mentioned. Virtually, the substantial pH differences in diverse GI tract regions make it challenging to combine a fixed pH-dependent hydrogel for both submucosal injection and wound healing. In addition, as one of the precursors of hydrogels, AA-NHS is synthesized by a two-step reaction in which the catalyst for grafting reaction is introduced, the possible residues of small catalyst molecules or byproducts inside AA/AA-NHS hydrogels are potentially to be toxic.

As described above, in addition to improving intraoperative elevation and support, the hydrogels applied during ESD has the significant advantage that it can be endowed with multiple functions through modification itself or combination with other components, which has led to the development of a new generation of multifunctional injectable hydrogels. Therefore, there are many other functional designs such as loading medicine [48] being applied to synthetic polymers in order to obtain multifunctional synthetic polymer hydrogels as SIMs in ESD. Figure 5D shows the difference between injectable drug eluting elastomeric polymer (iDEEP) hydrogels and sodium hyaluronate in submucosal elevation, and the corresponding submucosal elevation height changing curve and medicine release profiles could be found in Fig. 5E and F, respectively.

To sum up, the synthetic polymers mentioned above could be easily injected and in-site cured as well as be endowed with additional biological functions in a variety of ways, however, they are more or less likely to trigger unintended and undesirable biological reactions due to the poor biocompatibility of the synthetic polymers themselves or toxic monomer/initiator residues [49].

### Natural biopolymer hydrogels in ESD

Natural biopolymer is a kind of polymer matrix found in animals, plants and organisms, which exists in the form of high molecular weight compounds with the basic structure of linear long chains connected by repeated units, with common types of polysaccharides (starch, cellulose, alginate, hyaluronic acid, chitosan etc.), peptides (poly-l-lysine, poly-l-glutamic acid etc.) and proteins (collagen, gelatin, enzyme etc.). Based on their excellent bioactivity and biocompatibility, a growing number of scientists and clinicians began to use natural biopolymers to prepare hydrogels as SIMs for ESD in recent decades.

**Figure 6.**
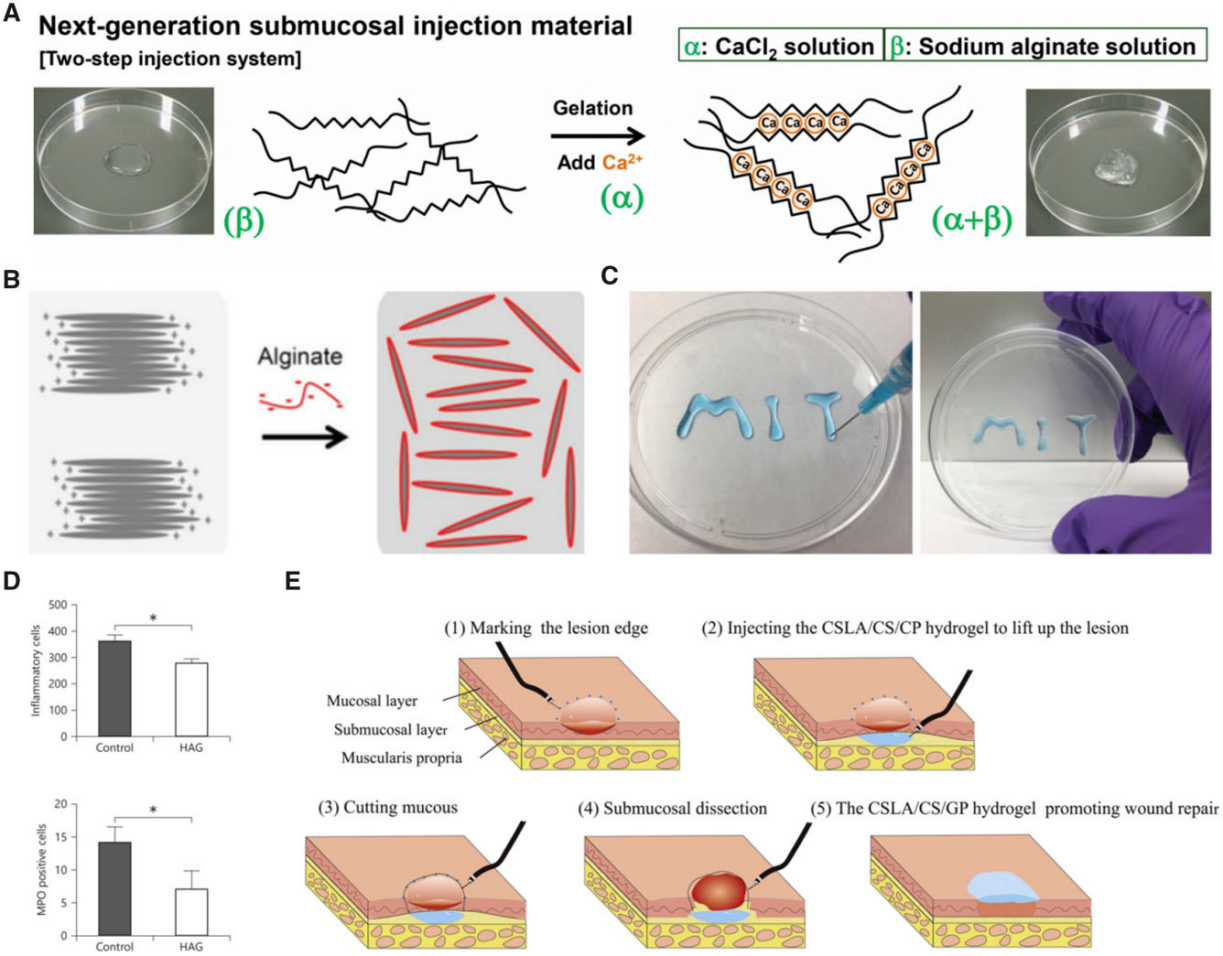
Various natural polymer hydrogels used in ESD. (**A**) Mechanism of sodium alginate gelling upon adding calcium ions (reproduced with permission from Elsevier). (**B**) Schematic illustration of exfoliated clay nanosheets (gray segments) by the interaction of their positively charged edges with anionic sodium alginate (red lines) (source article published under open access, CC-by license). (**C**) Feasible injection of endoscopically injectable shear-thinning hydrogels through a 25 gauge needle and the immediate reformation of a steady gel after injection (source article published under open access, CC-by license). (**D**) Comparing hexanoyl group-modified alkaline-treated gelatin-based membrane hydrogel group and control group, the former leads to a milder inflammatory response (reproduced with permission from Karger publishers). (**E**) The CSLA/CS/GP hydrogel can lift lesion and promote repair during endoscopic submucosal dissection procedures (reproduced with permission from Elsevier).

People first concentrated on single component natural biopolymer hydrogel. Chitosan first appeared on the stage in the early 20th century due to its eye-catching abilities of photo-cross-linking after azide and lactose moieties modification [50–52]. Then various types of chitosan thermosensitive hydrogels have been developed as a novel submucosal fluid cushion material for ESD based on different gelling agent, such as NaHCO_3_ [53], K_2_HPO_4_ [54] and β-glycerol phosphate disodium salt (β-GP) [33], to form a viscous gel in-site at body temperature. Due to that it could be quickly cross-linked through divalent cations aided by ionic coupling effects, which had been confirmed by eggbox model, sodium alginate became popular as an innovative endoscopic SIM. Hirose *et al.* [55] have proved a significant increase in the viscoelasticity of sodium alginate facilitated the formation of a highly viscous submucosal cushion by adding Ca^2+^ with a two-step injection procedure (Fig. 6A). Meanwhile, shear-thinning characteristic of the alginate-based hydrogel after composite Laponite make it possible to inject SIMs directly in one step (Fig. 6B and C) under the endoscope [56]. Later, with the needs of wound healing and tissue regeneration after ESD surgery, polypeptide with cell adhesion sites attracted the attention of researchers. Maeda *et al.* [57] found that hexanoyl (Hx:C6) group-modified alkaline-treated gelatin-based membrane hydrogel could induce proper healing by decreasing inflammation and fibrosis (Fig. 6D). Uraoka *et al*. [58] demonstrated that a novel temperature-responsive, biodegradable and injectable collagen sol could close colonic perforation holes after ESD though animal study. Hydrophobically modified Alaska-pollock gelatin microparticles designed by Ito *et al*. [59] were also used for solve delayed perforation. Going one step further, a fully synthetic and self-assembled peptide solution as SIM to elevate lesion without obvious tissue damage have already been proved by Nakata *et al*. [60] through a preliminary animal study.

As the complications of ESD surgery lead to longer operative time and more postoperative syndromes, SIMs require more biological functions, thus composite natural biopolymer hydrogel with multiple components has become a new research vane. Given that the clinical use of chitosan/glycerol phosphate (CS/GP) hydrogel is restricted by its sluggish gelation, low mechanical resistance and slight cytotoxicity from β-GP, collagen [61] was added to the thermosensitive hydrogel system to enhance tissue healing at the ulcers and subsequently lessen the possible cytotoxicity of β-GP. Similarly, a further improvement based on CS/GP system was developed, which was the addition of lactobionic acid-modified chitosan (CSLA) [62]. Moreover, due to the improved tissue adhesion and resistance to acidic environment, the CSLA/CS/GP hydrogels may possess the potential of promoting wound repair (Fig. 6E) [11]. In summary, natural biopolymer hydrogels offer advantages over the use of synthetic polymer hydrogels in many aspects, but the limitations of current research are also obvious. For example, in CS/GP-based hydrogels system, high concentrations of β-GP required for rapid gelation at physiological temperatures may lead to potential biological toxicity. It’s also worth mentioning that noncovalent molecular bonding interactions dominate inside natural biopolymer hydrogels, which are mainly weak physical interactions such as ionic interactions, hydrogen bonds, crystallization and hydrophobic interactions with typically fleeting nature, thus leading low strength and durability. In order to achieve more clinical functions such as mucosal protection, reducing perforation or bleeding, improving visibility during surgery and alleviating distress caused by tissue compression, the development of multifunctionally designed hydrogels is urgently needed now and in the future.

## Functional strategies of hydrogels for novel applications in ESD

According to the needs of ESD and its postoperative management, separation and elevation and anti-collapse are the most basic requirements for SIMs. Besides, the rich controllability and designability should be considered to endow hydrogels as SIMs more advanced performance and application. In recent decades, the relevant functional designs may concentrate in the following aspects.

### Optimizing physicochemical properties for better lesions’ elevation

**Figure 7.**
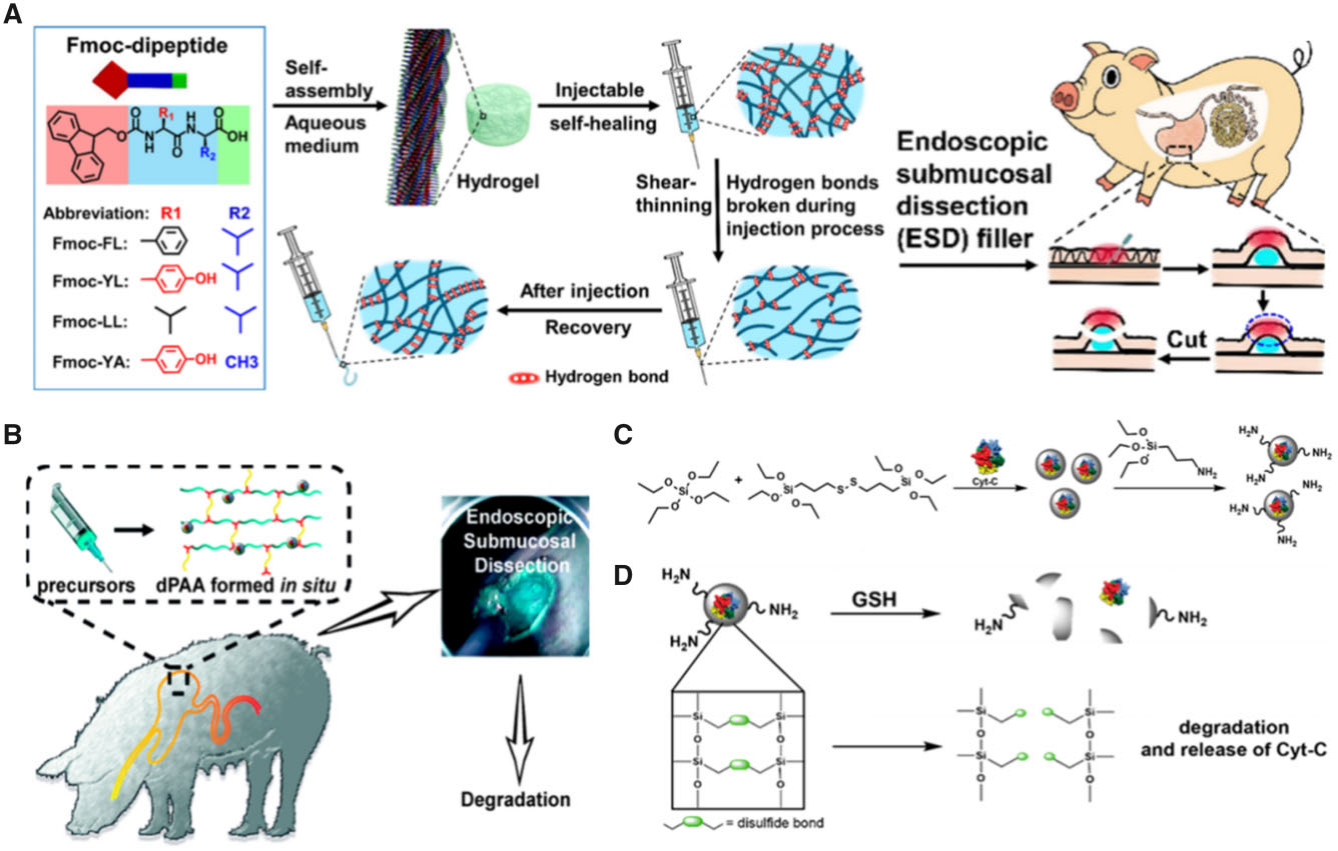
Self-healing, biodegradable hydrogel for elevating lesions and its novel functional design. (**A**) Schematic illustration of Fmoc-dipeptide self-assembly hydrogels with shear-thinning and self-healing properties (source article published under open access, CC-by license). (**B**) Schematic illustration of the injectable hybrid hydrogels with cell-responsive degradation for tumor resection (source article published under open access, CC-by license). (**C**) Scheme of the synthesis and functionalization of BNCs containing disulfide moieties in the framework and loaded with Cyt-C inside the silica capsule (source article published under open access, CC-by license). (**D**) Degradation of the BNCs upon exposure to cell-released glutathione (source article published under open access, CC-by license).

As previously stated, the key to a safe and successful ESD is intraoperative submucosal injection to raise lesions, which could not only divide the mucosal and muscular layers but also reveal the depth of invasion. Most existing hydrogels as SIMs for ESD rely on mechanical properties obtained after in-site gelling to maintain the elevation. Such properties, particularly in natural biopolymer hydrogels, are extremely fragile and may be mistakenly harmed during intraoperative lesion dissection, resulting in breakage and loss of supportive capacity. Under this circumstance, not only are abilities of injectable and gel-forming in-site needed, but also ability of self-healing is urged. Dipeptide self-assembled hydrogels have been investigated for possible biological uses in ESD due to their outstanding biocompatibility, bioactivity and tunable physicochemical properties that may be adjusted at the molecular level by tailoring amino acid sequences. Ren *et al.* [63] have successfully designed a series of dipeptides to form shear-thinning hydrogels with self-healing and tunable mechanical properties (Fig. 7A), which is supported by experimental studies and molecular dynamics simulations. In studies using mice and live mini-pigs, injectable dipeptide hydrogels were found as an excellent filler for ESD, which are superior to commercial clinical fillers in terms of duration, stiffness and inflammatory reaction.

The elevation effect should gradually fade after ESD surgery to accommodate the growth of new mucosa, which requires the injection material to be fully degradable. By incorporating breakable nanocapsules into a polyamidoamines-based hydrogel that contains disulfide, Alonci *et al.* [64] successfully developed a degradable hybrid hydrogel (Fig. 7B). The polyamidoamine-based hydrogel comprises breakable silica nanocapsules (BNCs) covalently attached to its network and capable of releasing biomolecules (Fig. 7C), and the hydrogel was degraded by the cells via glutathione secretion due to the existence of cleavable disulfide moieties (Fig. 7D). The same stimulation causes the silica nanocapsules to rupture, thus the entire hybrid material could be completely degraded.

Taking into account the increasing importance of intraoperative/postoperative wound management noticed by patients, it is outdated to focus solely on lesion elevation management. Thus more and more modified hydrogels with varied functional designs such as wet adhesion, hemostasis, interfacial compliance applied in ESD are arousing increasing interests in recent years.

### Promoting hemostasis and postoperative wound healing

**Figure 8.**
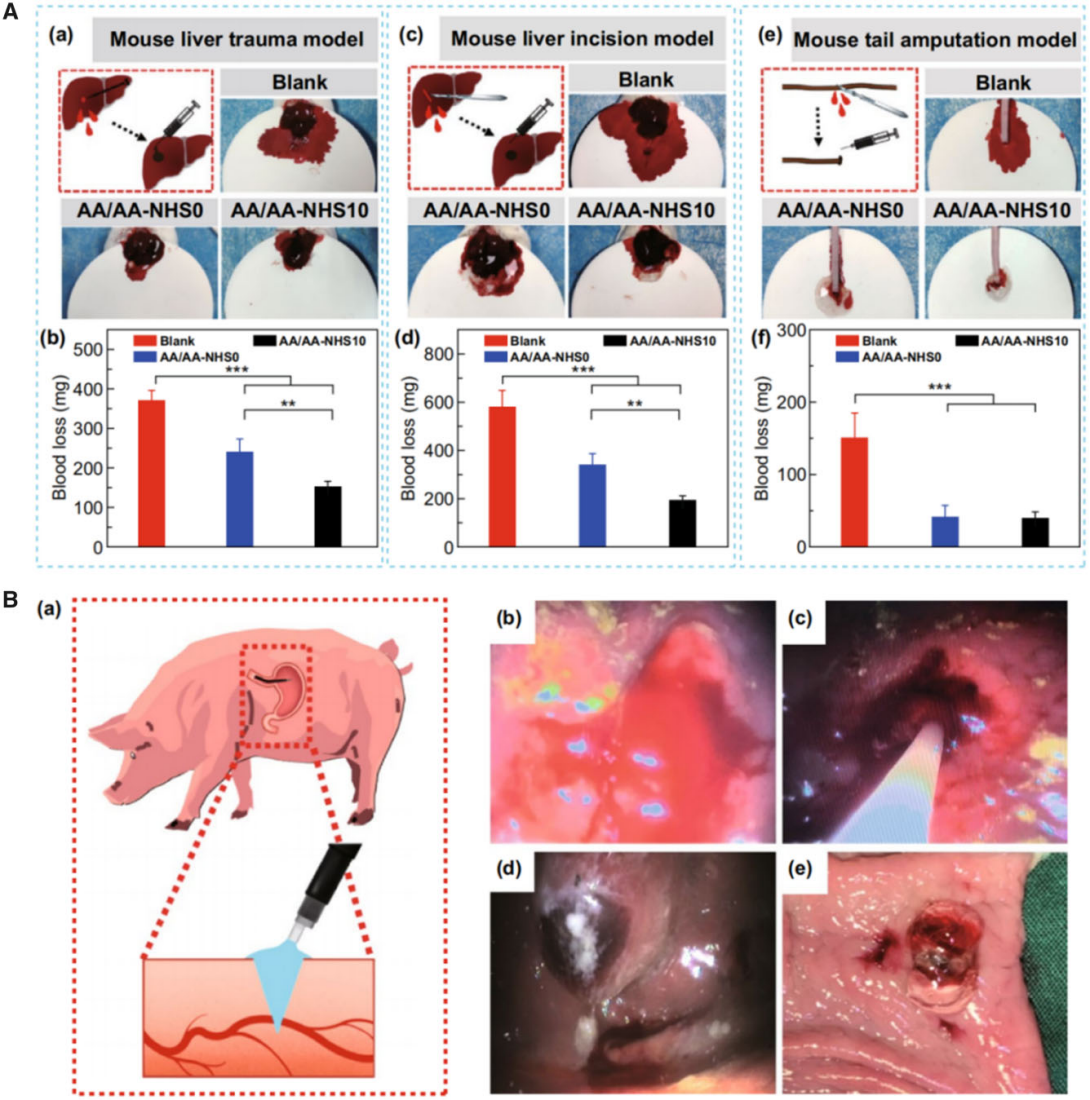
*In vivo/in vitro* hemostatic hydrogels. (**A**) (a) Schematic representation of the mouse liver trauma model; (b) quantitative data of blood loss (*n* = 6); (c) schematic representation of the mouse liver incision model; (d) quantitative data of blood loss (*n* = 6); (e) schematic representation of the mouse tail amputation model; (f) quantitative data of blood loss (*n* = 6). **P < 0.01, ***P < 0.005. (source article published under open access, CC-by license). (**B**) (a) Schematic representation of the investigation protocol diagram; (b) swine gastric bleeding model; (c) the AA/AA-NHS10 hydrogel was sprayed to the bleeding point with a spray tube through a gastroscope. The hydrogel strongly adhered to the gastric wall and stopped bleeding by forming hydrogel films *in vivo* (d) and *in vitro* (e) (source article published under open access, CC-by license).

Hemorrhage and perforation are the most serious complications associated with ESD, which may lead to hemorrhage shock, septic shock and even death. Moreover, intraoperative hemorrhage may also affect the operative visual field, further increasing the risk of perforation. Hence, hemostasis in time is necessary and common therapeutic strategies to halt bleeding include drugs, argon plasma coagulation, electrocoagulation and hemostatic clips. Due to its potential to reduce bleeding, diluted epinephrine (1:50 000–1:200 000) is frequently added to the submucosal injection fluid [65]. But the administration of epinephrine submucosally has the potential to cause systemic consequences, including life-threatening hypertension, cardiac tachycardia and intestinal ischemia [66]. Furthermore, multiple coagulation may cause coagulation syndrome and perforation [15]. Hemostatic clips may interfere with the surgeon’s procedure and perhaps cause muscular layer injury. With more and more hydrogels being used in the postoperative management of ESD, hemostatic function has become one of the key areas of functional design of hydrogels. For example, the AA/AA-NHS hydrogels [46] mentioned in ‘Synthetic polymer hydrogels in ESD’ showed excellent hemostatic behavior both *in vitro* liver injury models (Fig. 8A) and *in vivo* pig gastric bleeding/wound healing models (Fig. 8B). The good *in vivo* hemostatic capability of the AA/AA-NHS hydrogel was mainly due to the AA-NHS-containing hydrogel group had higher adhesive strength than the NHS-free group, thus creating a better seal on the wound, resulting in less blood flowing out.

For the reason of aiming at the lesions with a large lesion range and early cancerous area, the wound depth of ESD surgery is much deeper than endoscopic mucosal resection. In ESD surgery, surgeons judge the depth of the operation just according to their experience and the lesion’s size, it also means that in addition to mucosal layer injury even the most experienced surgeon may cause muscular layer injury. When the injury occurred, the injected hydrogels as SIMs should not only act as a hemostatic but also be able to promote wound healing continuously. For this reason, the hydrogels should have not only hemostatic properties but also other biological properties such as inducing muscle/mucosal layer tissue repair or regeneration, as well as compliance and adhesion to the wound interface.

**Figure 9.**
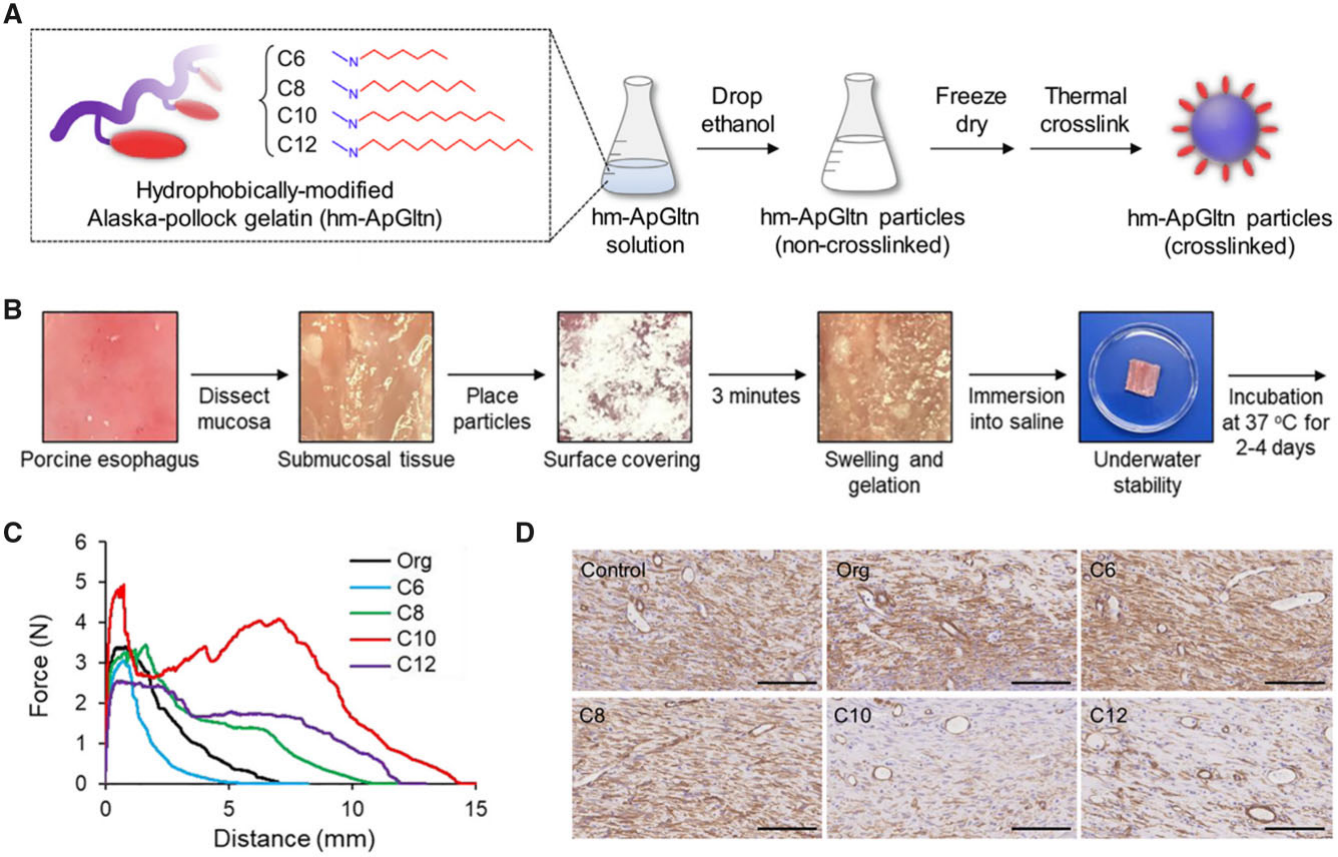
Bioadhesive, hemostatic and tissue regeneration-inducing hydrogels and their functional design. (**A**) Schematic illustration of synthesis of hm-ApGltn and preparation of cross-linked hm-ApGltn microparticles (reproduced with permission from Elsevier). (**B**) Underwater-adhesive stability test of hm-microparticles on esophageal submucosa tissues (reproduced with permission from Elsevier). (**C**) Force–distance curve of org- and hm-ApGltn microparticles treated with 6 h of thermal cross-linking (reproduced with permission from Elsevier). (**D**) Suppression of fibrosis in rat skin wound healing models by hm-microparticles (reproduced with permission from Elsevier).

Bioactive materials such as collagen play an important role in wound hemostasis and induced regeneration [67]. And the hemostatic process of collagen-based hydrogels is usually to gather platelets to form clots and then stop bleeding quickly [67]. At the same time, the presence of collagen endows composite hydrogel the ability to promote tissue regeneration. Furthermore, due to the possibility of intraoperative bleeding, the ability to adhere to the wet surface of the surgical site is important for the long-term retention of SIMs during ESD. Nishiguchi *et al*. [68] have successfully created a type of underwater-adhesive microparticle dressing using hydrophobically altered Alaska-pollock gelatin for GI tract wound healing (Fig. 9A). Through its contact with live tissues and cohesion force, gelatin’s hydrophobic alteration with aliphatic aldehydes improved its adhesion strength to stomach and esophageal submucosal tissues (Fig. 9B). In comparison to unmodified gelatin, optimal hydrophobic modification did significantly increase microparticle underwater stability and create a thick, integrated hydrogel layer on tissues (Fig. 9C). The aforementioned findings demonstrated the tremendous therapeutic potential of this gelatin-based microparticle wound dressing to speed up wound healing and prevent the development of new symptoms following ESD (Fig. 9D).

### Regulating immune response and anti-fibrotic effect

Existing studies have shown that the number of complications after ESD surgery is large and inevitable, especially when it comes to severe and cancerous lesions. In the pathophysiology of esophageal stricture, which is a typical symptom after extensive ESD for the treatment of esophageal cancer, immune cells and myofibroblasts play a critical role. The lack of epithelial barrier function after ESD surgery triggered immune cell recruitment, resulting in an infiltrating inflammatory response [69]. Then, fibrosis is caused by the transformation of fibroblasts and pericytes into α-smooth actin-expressing myofibroblasts [70]. Consequently, submucosal fibrosis is related with the deterioration of muscularis mucosae and the infiltration of muscularis propria, resulting in esophageal strictures.

**Figure 10.**
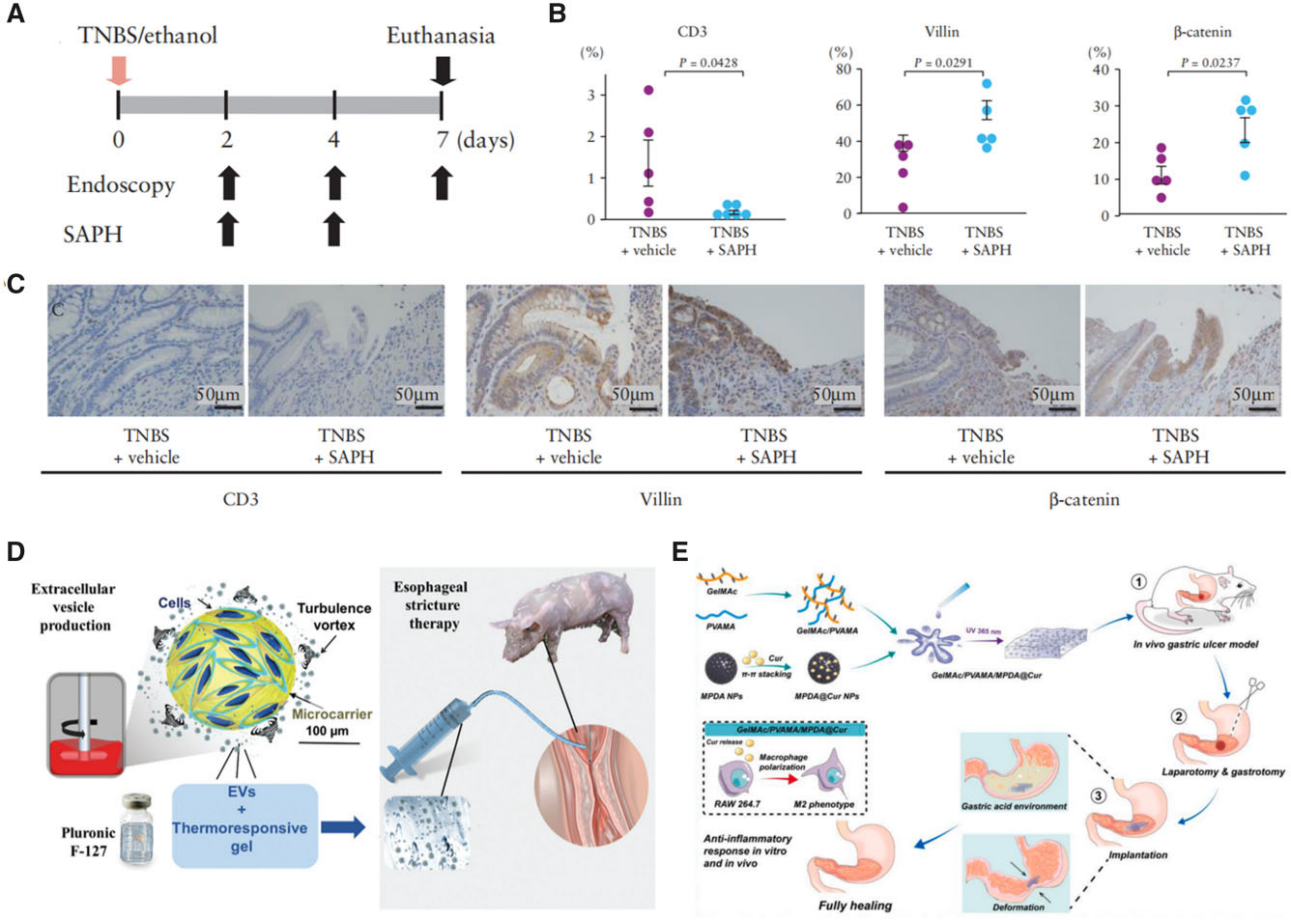
Anti-inflammation and anti-fibrosis hydrogel and its functional design for postoperative stricture and ulcer treatment. (**A**) Timeline of the SAPH on trinitrobenzene sulfonic acid (TNBS)-induced colonic ulcers. (**B**) The number of CD3, villin and β-catenin-positive cells in colon cross-sections of vehicle- or SAPH-treated TNBS rats at Day 7 after TNBS/ethanol injection. (**C**) Representative immunohistochemical localization of CD3, villin and β-catenin in colon cross-sections of vehicle- or SAPH-treated TNBS rats at Day 7 after TNBS/ethanol injection (hematoxylin and eosin staining) (reproduced with permission from Oxford University Press). (**D**) Schematic representation of the experimental approach consisting of producing EVs by turbulence stimulation from swine ADSCs cultured on microcarriers in bioreactors. ADSC EVs were combined with the pluronic F-127 thermoresponsive hydrogel and administered at <19°C (below the gel transition temperature) at post-resection esophageal lesion gelling *in situ* at body temperature (reproduced with permission from Royal Society of Chemistry). (**E**) Schematic illustration of the GelMAc/PVAMA/MPDA@Cur hydrogel for improving gastric ulcer healing via regulating immune response (reproduced with permission from Elsevier).

The self-assembling peptide hydrogel (SAPH), a completely synthetic peptide solution designed to substitute collagen, has been recently utilized to enhance mucosal regeneration in iatrogenic ulcers after ESD (Fig. 10A). The experimental results showed that in the rat model, SAPH administration successfully inhibited colonic damage, decreased inflammatory cytokine expression, and increased wound healing-related factor expression (Fig. 10B and C) [71]. To prevent the formation of stricture after ESD in a porcine model, Coffin *et al*. [72] looked at the connection between Pluronic^®^F-127, a thermoresponsive gel, with extracellular (nano) vesicles (EVs) formed from pig adipose tissue-derived stromal cells (ADSCs). The use of EVs plus gel following prolonged esophageal endoscopic resection was successful in avoiding stricture development while also having an anti-fibrotic impact (Fig. 10D). Since esophageal stricture is the most problematic delayed syndrome after prolonged superficial cancer removal by endoscopy, this nano-therapy may be of relevance in addressing an unmet medical need.

Another serious complication, artificial ulcers produced after ESD procedure, continues to afflict millions of clinical patients, and the ideal healing of it is still hard to achieve. The root cause is that the intrinsic peristole, acid situation and ultra oxidative stress in the stomach ulcer environment have a significant impact on the therapeutic success of standard medicine. For addressing the application challenge of hydrogel-based SIMs in the above clinical setting, Zhang *et al.* [73] reported a hybrid hydrogel system made up of catechol motif-modified methacrylated gelatin (GelMAc) and methacrylated poly (vinyl alcohol) (PVAMA), in which GelMAc significantly improved adhesion properties and biocompatibility while PVAMA efficiently provided stability and mechanical strength (Fig. 10E). In addition, as the drug carrier, mesoporous polydopamine nanoparticles were produced and enclosed in the aforementioned hybrid hydrogel system via π-π stacking and hydrogen bonding interactions. The final sample has been demonstrated to exhibit exceptional stability on stomach tissue even under acid (pH 2) and oxidative stress (H_2_O_2_) conditions. The sample’s slowly released curcumin was capable of triggering M2 polarization of macrophages. In an *in vivo* stomach ulcer model, the produced sample significantly enhanced gastric ulcer healing by suppressing the pro-inflammatory response and relieving oxidative stress as well as accelerating reepithelization and angiogenesis.

All in all, in view of various intraoperative or postoperative complex sequelae and complications associated with ESD surgery, in addition to playing the role of lesion elevating mentioned above, hydrogel-based SIMs is expected to solve a series of clinical nursing problems in combination with the regulation of immune response and anti-fibrosis and other biological functions. Therefore, the injectable hydrogel materials with new biological functions will show bright application prospects in ESD field.

## Conclusions and prospect

The current hydrogels used as SIMs for ESD surgery possess good physical attributes, but their biological functions have been largely ignored. These hydrogels provide a stable and safe cushion for the surgeon to work on, but do not address the critical issue of promoting postoperative wound healing and preventing postoperative stenosis. To facilitate the reader access to relevant information, the hydrogels in this article and their corresponding source literature have been listed in Table 1. In conclusion, the perfect hydrogels as SIMs used for ESD surgery should have the following outstanding advantages: (i) convenient syringeability and lasting intraoperative elevating effects without rapid collapse. (ii) Potential for handling various postoperative complications: Hydrogels with diverse biological functions could help stop bleeding and promote healing during or after surgery by providing a barrier between the wound and the corrosive digestive fluids, resist lumen stenosis by regulating immune response, anti-fibrosis and antibacterial. (iii) Improve visibility: maintaining a clear visual field without bleeding during ESD is important for achieving better *en bloc* resection rates. Hydrogels can also improve visibility during surgery by preventing blood and other fluids from obscuring the lesion's visual field, which could help surgeons make more accurate cuts and remove lesions more effectively. (iv) Providing conformability, convenience, and patient comfort while biodegrading safely in human tissue after a period of time. (v) Cost-effective and easily accessible, which would allow for widespread use and availability of these materials, making ESD surgery safer and more accessible to patients who need it.

**Table 1.** The hydrogels used in endoscopic submucosal dissection and their corresponding source literature

Hydrogels in endoscopic submucosal dissection	Types of the raw material		Types of the injection way		Types of the function	
	Synthetic polymer	Natural biopolymer	Via shear-thinning	As precursor	Physical	Biological
Abbreviation of hydrogels (Ref)	PLGA-PEG-PLGA hydrogel [45, 46], AA/AA-NHS hydrogel [47], iDEEP hydrogel [48], dPAA hydrogel [64], GelMAc/PVAMA/MPDA@Cur hydrogel [73]	Alg hydrogel [55, 56], HAG hydrogel [59], CSLA/CS/GP hydrogel [61, 62], Fmoc-YL hydrogel [63], hm-ApGltn hydrogel [68], SAPH hydrogel [71], PF-127/EV hydrogel [72]	AA/AA-NHS hydrogel [47], iDEEP hydrogel [48], dPAA Hydrogel [64], GelMAc/PVAMA/MPDA@Cur hydrogel [73], Alg hydrogel [55, 56], HAG hydrogel [59], CSLA/CS/GP hydrogel [61, 62], Fmoc-YL hydrogel [63]	PLGA-PEG-PLGA hydrogel [45, 46], hm-ApGltn hydrogel [68], SAPH hydrogel [71], PF-127/EV hydrogel [72]	PLGA-PEG-PLGA hydrogel [45, 46], Alg hydrogel [55, 56]	AA/AA-NHS hydrogel [47], iDEEP hydrogel [48], dPAA Hydrogel [64], GelMAc/PVAMA/MPDA@Cur hydrogel [73], HAG hydrogel [59], CSLA/CS/GP hydrogel [61, 62], Fmoc-YL hydrogel [63] hm-ApGltn hydrogel [68], SAPH hydrogel [71], PF-127/EV hydrogel [72]

However, with the aid of existing multifunctional hydrogel-based SIMs to perform ESD procedure, patients and surgeons of GI cancer still confront many serious challenges, especially when the lesions are deeply infiltrated or located on the surgical scar. When the lesions were heavily infiltrated, submucosal injection would result in insufficient submucosal uplift, leading a narrow gap between the mucosal layer and the muscular layer. At this time, the muscular layer will be easily damaged when the electrotome is operated inside, which may cause intraoperative perforation, or delayed perforation of muscular layer fissure damaged by postoperative eating. In addition, the submucosa is not easy to separate when the lesions are deeply infiltrated, and in this case, the submucosal blood vessels are more abundant and prone to bleeding. Perforation in above cases may lead to peritonitis, septic shock and even death. In addition, scar tissue is denser than normal tissue, making it more difficult to create a stable cushion for dissection. As a result, the risk of perforation and bleeding increases, and the procedure becomes more challenging for the surgeon. Hence, considering the challenges associated with performing ESD on an infiltrated lesion or scar, the hydrogels should meet these special needs, including higher mechanical strength and better hemostasis properties.

To address these challenges, researchers are exploring the combination of hydrogels with functional materials, such as small-molecule drugs or novel nanomaterials, to achieve the desired clinical multiple functions for postoperative management of ESD. On the one hand, the introduction of small-molecule drugs is one of the most direct means to improve postoperative management, and has a significant effect on the treatment of various complications after ESD surgery. Therefore, it is an important development direction of hydrogel-based SIMs to achieve sequential controlled release and functional collaboration through proper combination of drug molecules and hydrogels. On the other hand, due to the versatility of nanomaterials, such as anti-inflammation, hemostasis, targeted anti-cancer, antibacterial effect, the combination of hydrogels and nanomaterials could greatly expand their applications in the field of biomedical engineering. Through simple and flexible selection of different types of nanomaterials/hydrogels, or adjusting the physicochemical interaction between nanomaterials and hydrogels, some specific properties far beyond that of traditional hydrogels can be realized, at the same time, providing economic cost and accessibility strategy for SIMs.
